# Financial and economic analyses of the impact of cattle mastitis on the profitability of Egyptian dairy farms

**DOI:** 10.14202/vetworld.2020.1750-1759

**Published:** 2020-09-02

**Authors:** M. F. Azooz, Safaa A. El-Wakeel, H. M. Yousef

**Affiliations:** 1 Central Laboratory for Evaluation of Veterinary Biologics, Abbasia, Cairo, Egypt; 2 Animal Reproduction Research Institute, 5 Garden Center Research Street Haram, Giza, Egypt; 3 Department of Infectious Diseases, Faculty of Veterinary Medicine, Cairo University, Giza, Egypt

**Keywords:** clinical mastitis, economic impact, financial losses, subclinical mastitis

## Abstract

**Aim:**

This study aimed to evaluate and quantify the different factors affecting the costs of mastitis in cattle, to quantify the annual and monthly financial losses attributed to mastitis, and to estimate production losses using average linear scores found on The Dairy Herd Improvement Association somatic cell count (SCC) sheets and bulk tank SCC per lactation number.

**Materials and Methods:**

All data (bovine mastitis-associated costs and expenditures in Egyptian dairy herds) were analyzed using SPSS/PCT, 2001. A partial budget technique using spreadsheet software and the general linear model procedure was used to analyze the productive and financial measures.

**Results:**

Mastitis was present in 57.1% of cows (240/420), while clinical mastitis (CM) was present in 19% of them (80/420). The geometric mean of SCC/ml in bulk tank milk samples of 150 cattle dairy farms was 556.3×10^3^. The annual subclinical mastitis (SCM)-related economic loss was 21,933,258.6 LE, and the two most important cost components were the subsequent decrease in milk production and quality premium losses (93% and 7% of SCM costs, respectively). The quality premium loss was 1,369,602.1 LE. On the other hand, the annual economic loss due to decreased milk production as a result of SCM was 20,563,656.5 LE. The total cost of 80 CM cases, including the failure and preventive costs, was 1,196,871.4 LE, including 1,169,150.4 LE failure costs (106,336.0 LE in direct costs and 1,062,814.4 LE in indirect costs) and 27,721.0 LE preventive costs. The average cost per CM case was 28,760.9 LE, including veterinary time and consultation fees of 250.0 LE (1%), labor 562.5 LE (2%), premature culling 736,000.0 LE (77%), decreased milk production 4085.18 LE (13.7%), discarded milk 185.3 LE (1%), and drugs and treatments 328.9 LE (1%). The total costs of CM (expenditures) extra control and preventive measures, including the diagnosis of CM for 80 cows annually in 20 Egyptian dairy farms were 27,721.0 LE, representing 346.5 LE or 1% of the total cost of CM cases. The cost of monitoring and diagnostic measures was 8635.2 LE, representing 107.9 LE or 1% of the total cost of a case of CM.

**Conclusion:**

The method used for cost estimation, in this study, is highly adaptable to individual cattle farms and had a major role in assessing specific control and management measures. The concepts described in this paper help to improve our understanding of the full economic impact of clinical and subclinical mastitis in cattle in Egypt. Assessing the economic losses from mastitis to determine the economic costs and losses occurring in Egyptian dairy farms is critical for encouraging farmers to acknowledge the scale of the problem and implement effective management practices aimed at improving mastitis control and reducing the associated costs.

## Introduction

Bovine mastitis is regarded as one of the most economically damaging diseases in the dairy industry globally [1-3]. There is a need for accurate information about the actual costs of mastitis to establish appropriate economic incentives to prevent and treat it. Such information would help to evaluate the viability of preventive measures concerning a single dairy cow, a whole herd, as well as the whole dairy sector. The costs of bovine mastitis can be illustrated and divided into two main categories: Production losses and control-related expenditures [4]. Economic losses due to mastitis can be defined as a reduction of output due to this disease and an absence of benefits that would otherwise be accrued in the absence of mastitis. The former of these can be exemplified by milk that has to be discarded following treatment with antibiotics, while the latter could refer to milk that is never produced as a result of this disease. Expenditures are additional inputs needed to reduce losses due to mastitis, either by reducing the impact of mastitis, such as treating mastitis-affected cows or by preventing mastitis losses from occurring, as in the case of investments in preventive measures. Mastitis costs are also classified into two main categories: Those occurring directly and indirectly [5]. Direct costs consist of veterinary services, diagnostics, treatments, additional labor requirements, and discarded milk (during the course of treatment). Indirect costs are defined as those that are not always obvious to the milk producer, also known as hidden costs. Indirect losses due to subclinical mastitis (SCM) are not well recognized by many farmers, but include reduced milk yield, premature culling losses, and reduced quality premiums [6]. It is generally accepted that SCM accounts for the majority of the economic costs of mastitis. Education on this matter is critical because unrecognized indirect losses would be a reason for the difficulty in implementing mastitis control measures. Mastitis can involve two main forms: Clinical and subclinical forms. From an economic perspective, for many cattle farms, SCM is thought to be the most economically important type of mastitis because of the long-term effect of chronic infections on total milk yield. It causes substantial damage to the milk secretory cells and results in reduced milk production, changes in milk composition and quality, and also a shortened lifespan of diseased animals [7].

SCM has been shown to be responsible for most of the economic losses due to mastitis, with a reduction in milk production being the main factor. It has been reported [8] that the economic losses caused by SCM in the United States dairy industry exceed $1 billion annually. Typically, SCM costs£140 ($226) per case; the decrease in milk production, preventive and control measures, and premature culling costs was the top three cost categories. Within high bulk tank somatic cell count (BTSCC) herds, the cost of SCM was £217 ($351) per case; within low-BTSCC herds, the typical cost of SCM was £68.90 ($111.40) per case [9]. Several studies have taken all of the direct and indirect costs of clinical mastitis (CM) into consideration and produced average figures of $179 [10], $254 [11], $266 [12], $444 [13], and $518 [14] for the cost of a case of CM. Quantification and measurement of the economic losses of cattle clinical and subclinical mastitis are mainly based on two approaches: Analysis based on farm livestock productivity data and data analysis through partial budgeting and dynamic simulation. The economic losses due to mastitis can be estimated and assessed by various methods. Partial budgets are generally easy to design and play an important role in providing an accurate estimate of the economic impact of mastitis in the dairy sector; they can be used by advisors and farmers to support decisions concerning animal health management [5,6,15,16].

Against this background, the main objectives of this study are to quantify and assess the annual herd economic losses caused by bovine clinical and subclinical mastitis within large Egyptian dairy herds. These objectives are often achieved using the following approaches: Quantification, determination, and evaluation of the incidence and prevalence of ­clinical and subclinical mastitis in the populace, which is essential for estimating its actual cost to the dairy industry. In addition, identification of the preventive and therapeutic measures undertaken is required. It is usually easy to calculate the expenditure on mastitis control, through collection and analysis of various sources of data, substances and records, the scientific literature, regulations, reviews, reports, suggestions, and recommendations, as well as websites and the internet. Evaluation and quantification of the different factors affecting CM costs are also important, including estimation and evaluation of selected factors associated with current expenditures for mastitis treatment and control and mastitis-associated output losses, including factors related to drugs, discarded milk, veterinary services, labor, product quality, diagnostics, reduced milk yield, and culling. Moreover, there is a need for quantifying annual and monthly financial losses attributable to SCM and estimating production losses using average linear scores found on The Dairy Herd Improvement Association (DHIA) SCC sheets and BTSCC per lactation number.

## Materials and Methods

### Ethical approval

The approval from the Institutional Animal Ethics Committee to conduct this study was not required as no invasive procedure on the animals was performed. However, this study was conducted in accordance with the standards of Institutional Animal regulations and Ethics of Animal Reproduction Research Institute, Haram, Giza, Agricultural Research Center.

### Animals, study design, and study area

A cross-sectional study was conducted from 2017 to 2019 on field data collected during field Ph.D. study entitled “Epidemiological studies on bovine mastitis in Egypt in Delta Region, Alexandria Road and the upper and lower Egypt Districts.” Samples were collected from a total number of 170 private, dairy cattle farms with total population number 9810, lactating cows belonging to Cairo, Giza, Qalubia, Sharkia, Monofia, Alexandria, Behera, Dakahlia, Bensulf, Fayoum, Sohag, and Aswan and Asuit governorates.

The study animals were dairy cattle originating from intensive dairy herds. The intensive dairy farms contained cattle from small, medium, and large dairy herds. The farms were selected based on the availability of lactating cows within the farm and the owners’ willingness to participate. Economic data were obtained from relevant records stored at those farms. Cow parity was defined as follows: Primiparous (first-lactation) and multiparous (second lactation or later). Cows were machine-milked 3 times a day in milking parlors. Teats of cows were sanitized by dipping them in a 0.5% iodine teat dip before and after milking. The age of the cows was between 2.5 and 6 years in different stages of lactations. A total of 420 composite milk samples and 1680 quarter milk samples were collected from 420 lactating animals at 20 cattle dairy farms, having 1178 dairy cows, among which 80 showed clinical signs of mastitis. Overall, 150 bulk tank milk samples were collected from 150 cattle dairy farms, derived from a total of 8667 dairy cows. The udder was examined clinically in accordance with methods described previously [17], and 340 apparently healthy lactating animals were examined for indirect estimation of the SCC using the California Mastitis Test, in accordance with previously described methods [18-20]. The individual composite milk samples and bulk tank milk samples were automatically examined for SCC using Bently soma count 150 (Bently, USA), in accordance with previously described methods [21].

### Questionnaire survey and data collection tools for the economic model

All variables needed for the economic model were determined in line with previously described methods [7]. The main information collection tools used to acquire records on bovine mastitis-associated costs and expenditures in Egyptian dairy herds were a questionnaire and personal interviews with farm owners conducted in accordance with a previously described design [11,22]. The questionnaires and supporting material were established based on data from the literature and expert estimates. The questionnaire was designed mainly with questions on three categories. The first focused on general farm characteristics and also the status of mastitis. The second part addressing the bonus/penalty system was tabulated into two main parts: The first one dealing with the bonus system and the other one concerning the penalty system. The farm owners were asked about some aspects, statements, and declarations concerning the bonus and penalty systems. Farm owners were then asked about how large the bonus or penalty (depending on the questionnaire version) for various BTSCC thresholds (400,000, 350,000, 300,000, 250,000, and 200,000 cells/ml) would have to be for them to alter their farm management so as to remain below that threshold. The third category concerned the implementation of current mastitis management measures (milkers wearing gloves, teat dipping preparations, milking cows in groups based on SCC, dry cow therapy, culling policy, and diagnostics).

### Economic framework for the current study

The mastitis economic framework was carried out in accordance with previously described methods [7,23-26]. A cross-sectional study was designed to collect data on factors previously identified in studies to have an impact on mastitis-associated costs. Selected factors were those dealing with current expenditures for mastitis treatment and control and mastitis-associated output losses, and included factors related to drugs, discarded milk, reduced milk yield, veterinary services, labor, product quality, diagnostic, culling, materials, and investments. For each mastitis cost component, equations were formulated to ­estimate the cost over a year for a given herd.

### Quantification of veterinary services, time, and consultation fees

Veterinary services, time, and consultation fees were quantified in accordance with previously described methods [7,24,26]. These were quantified by determining the number and proportion of CM cases attended by a veterinarian, average cost per veterinary visit, and costs and fees of professional advice concerning herd mastitis-related issues.

### Estimation of drug and treatment costs

Assessment of drug and treatment costs was carried out in accordance with the methods described previously [24,26,27]. To measure the economic costs of drugs used for CM treatment, we took into consideration for every farm the number of CM cases over a year, the proportion of severe cases, the proportion of mild and moderate CM cases that were treated, the average number of cases per day treated, the frequency of therapeutic administrations per day, and the cost of therapeutics per administration.

### Economic losses due to discarded milk

The economic losses from discarded milk were calculated in accordance with the methods described previously [15,23,24,26]. The cost of discarded milk can be assessed from the calculation of the number of CM cases, average duration of treatment, the proportion of CM cases that received treatment, withdrawal time of the used drugs, duration of discarding milk in CM cases that are not treated, average daily milk production of a cow, costs of production of 1 kg of milk, and the proportion of discarded milk fed to calves. While the cost of milk replacement reflects the value of discarded milk that is used to displace purchased milk replacement, this value reached 85% of its cost, in accordance with methods described previously [13]. Based on this approach, discarded milk used to feed calves was assumed to have a value of 5.98/kg of liquid milk. Thus, the economic loss of a kilogram of discarded milk was equal to 7.04-5.98 LE, or 1.06 LE of lost potential value.

### Extra labor costs for mastitis

Labor costs were quantified in accordance with the methods described previously [4,7,23,24,26]. To calculate and measure the losses and costs of labor associated with mastitis treatment, the number of CM cases, mean time spent working on a CM case, and mean hourly fees were taken into account.

### Decrease in milk production

Milk production yield losses were quantified in accordance with the methods described previously [23]. Subclinical milk production losses were assessed using the input of the farms’ BTSCC, in accordance with methods described previously [23], and total production losses due to CM were calculated in accordance with other previously described methods [11,26] by multiplying the number of CM cases by the associated production losses per month.

### Quantification of quality premium loss and penalties

Quality premium loss and penalties were quantified in accordance with the methods described previously [15,23,26]. All economic costs dealing with premium loss and penalties at the farm level are easily calculated from statements of the maximum available SCC premium, currently received SCC premium, the potential premium difference, and finally the number of hundredweights shipped in the previous month multiplied by the potential premium difference to obtain the current monthly premium opportunity.

### Quantification of premature culling costs

Premature culling costs were calculated in accordance with previously described methods [15,24,26,28]. These can be quantified by estimating the number of primiparious and older cows that were culled or died due to CM or SCM, the costs of rearing or buying a first-lactation cow, the difference in milk production between primiparous and multiparous cows, the money received for meat or milk when selling a cow, and the money spent on carcass disposal for dead cows.

### The costs of diagnostic and monitoring measures

The costs of diagnostic and monitoring measures could be calculated in accordance with previously described methods [24,26]. This involved quantifying the number of samples collected in a year for CM and the costs per sample.

### The costs of mastitis control programs

The costs of control measures were quantified in accordance with previously described methods [24,26,27]. The costs of mastitis control include expenditures that can be calculated directly from expenses for pre- and post-milking teat disinfection, use of gloves for milking and dry cow therapy, other measures such as milking machine maintenance and use of towels, or calculated in accordance with standard treatment and prevention costs and from the amount of labor for monitoring and implementing treatment, prevention, and other expenditures.

### Statistical analysis

All data (bovine mastitis-associated costs and expenditures in Egyptian dairy herds) were analyzed using SPSS/PCT, 2001, in accordance with previously described methods [29]. A partial budget technique using spreadsheet software and the general linear model procedure was used to analyze the productive and financial measures.

## Results

Clinical and subclinical CM was found in 57.1% of cows (240/420). CM was found in 19% of cows (80/420). The geometric mean of SCCs/ml in BTM samples of 150 cattle dairy farms was 565.3 × 10^3^.

Analyzing the economic losses due to SCM revealed that the annual SCM economic loss was 21,933,258.60 LE (Table-1). It also showed that the two most important cost components were the subsequent decrease in milk production and quality premium losses (93% and 7% of SCM costs, respectively). Assessing the quality premium loss for 150 cattle dairy herds with a total of 8867 cows gave a value of 1,369,602.12 LE. The annual economic loss due to decreased milk production as a result of SCM was 20,563,656.5 LE.

**Table-1:** The overall economic losses of cattle mastitis and supply chain management.

Mastitis losses	Parameter	Cost/LE/Year
Subclinical mastitis losses	Milk yield losses	20,563,656.4826
	Quality premium	1,369,602.12
Clinical mastitis losses	Medicine costs	45,000
	Preventive costs	27,721
	Cost of discarded milk	100,172
	Veterinary services cost	20,000
	Extra Labor cost	45,000
	Control measures costs	27,721
	Cost of premature culling	736,000
	Milk yield losses	326,814

The quantification of the economic losses due to CM for 80 CM cases from 20 dairy cattle farms revealed that the total cost of these cases, including the failure and preventive costs, was 1,196,871.4 LE, including 1,169,150.4 LE for failure costs (106,336 LE for direct costs and 1,062,814.4 LE for indirect costs) and 27,721 LE for preventive costs.

The quantification of the veterinary time and consultation fees for CM revealed that the mean cost of veterinary time and consultation fees was 250 LE per case. The cost of veterinary time and consultation fees represented 1% of the total costs per CM case. The mean cost of labor was 562.5 LE per CM case (Tables-1 and 2). Labor cost represented 2% of the total costs per case.

**Table-2:** Estimated partial cost per case of cattle mastitis on several Egyptian dairy farms.

Farm	Average medicine costs/LE	Average No., days for discarded milk	Average production for cow discarded	Milk price/LE	Total cost of discarded milk/LE	Labor cost/case/LE	Veterinary service and consultation cost/case/LE	No. of clinical cases	Total costs/Year
F01	415	6	27	1.06	171.70	562.50	250	4	5596.80
F02	252	6	32	1.06	203.20	562.50	250	2	2536.04
F03	316	6	29	1.06	184.40	562.50	250	3	3938.80
F04	168	6	36	1.06	228.90	562.50	250	2	2418.90
F05	390	6	40	1.06	254.40	562.50	250	4	5827.60
F06	375	6	24	1.06	152.60	562.50	250	1	1340.50
F07	324	6	37	1.06	235.30	562.50	250	5	6859.10
F08	297	6	22	1.06	139.20	562.50	250	4	4994.80
F09	373	6	35	1.06	222.60	562.50	250	3	3570.30
F10	355	6	21	1.06	133.60	562.50	250	2	2602.10
F11	357	6	36	1.06	228.90	562.50	250	4	5593.80
F12	256	6	28	1.06	178.00	562.50	250	3	3739.70
F13	391	6	34	1.06	216.20	562.50	250	6	8518.40
F14	343	6	22	1.06	139.90	562.50	250	3	3886.30
F15	373	6	29	1.06	184.40	562.50	250	6	8219.60
F16	316	6	23	1.06	146.20	562.50	250	5	6373.90
F17	328	6	36	1.06	228.90	562.50	250	5	6847.30
F18	375	6	32	1.06	203.80	562.50	250	9	12402.20
F19	362	6	31	1.06	197.20	562.50	250	6	8229.90
F20	314	6	21	1.06	133.60	562.50	250	3	3780.00

The quantification of premature culling costs due to CM was also performed. The cost of premature culling due to CM was 736,000 LE for 80 CM cows per year, as shown in Table-1. The total culling in 20 cattle dairy farms with 1178 cows was 34 cows (2.88%). Future culling and replacement loss represented 23,000 LE, constituting 77% of the total cost of a case of CM.

The total economic loss due to decreased milk production as a result of CM for 80 CM cases was 326,814 LE. Decreased milk production as a result of CM represented 4085.18 LE of the total cost of a CM case.

The total cost of CM (expenditures) extra control and preventive measures, including the diagnosis of CM for 80 cows annually in 20 Egyptian dairy farms, was 27,721 LE, as shown in Table-1. (Expenditures) extra control and preventive measures represented 346.5 LE per clinical case or 1% of the total cost.

The mean drug and treatment cost of 80 CM cases were 328.85 LE per case, as shown in Tables-1 and 2. Therapeutic costs, which are often the most visible cost to producers, represented only 1% of the total costs of a case of CM. The mean discarded milk cost of CM was 185.25 LE, revealing that the discarded milk cost of CM represented only 1% of the total cost of a case of CM.

## Discussion

The main goals of this study were to evaluate the economic costs of cattle CM and SCM in Egyptian dairy farms and to determine the distribution among the various cost components. Mastitis is regarded as the most common disease of dairy cows and causes great economic loss to the dairy industry [7,30,31]. The distribution of costs associated with CM varies from farm to farm and region to region, depending on the mastitis control strategy and environmental conditions. The distribution of costs can indicate the most appropriate way of establishing a mastitis control program. The total economic costs are not the most important figure [32].

The indirect losses of SCM do not seem to be well known and visible among many farmers. It is mostly accepted that SCM is responsible for the majority of economic losses due to mastitis [33]. The two most easily identifiable costs related to SCM are milk quality price penalties and loss of milk production.

The annual economic loss due to SCM was 21,933,258.6 LE. The two most important cost components were the subsequent decrease in milk production and quality premium losses (93% and 7% of SCM costs, respectively).

The results of assessing quality premium losses for 150 cattle dairy herds with a total of 8867 cows gave a value of 1,369,602.12 LE. As shown in the tables, these quality premiums are mostly based on SCC because SCC reflects the inflammatory process and thus the changes in milk composition. Most milk purchasers select milk with low SCC and offer financial incentives to farmers for high-quality milk. In the dairy industry, all over the world, penalty and premium programs have been designed to produce incentives and motivations for dairy producers to boost milk quality [34]. Most of these programs are focused on bulk tank milk quality, for example, SCC [35]. Premium payments encourage farmers to provide high-quality milk without disrupting the milk supply chain [34]. High-SCC milk is not desirable for dairy processors because it reduces the shelf life of dairy products and reduces the quality and quantity of milk protein, thereby reducing cheese yield. All of these changes result in a less valuable product and also confer inconsistent quality on the product after a shorter period of storage. This has a great effect on the consumers’ attitude to the product and thus also on their willingness to buy the product at a high price in the future. It is not the cell count by itself that is important, but the association of SCC with the changes in composition. Quality premiums are an excellent opportunity for farmers to increase the marginal profit of their farms because they provide one of the few incentives for farmers to significantly impact the value of the milk that they receive [27].

On the other hand, the annual economic loss due to decreased milk production as a result of SCM was 20,563,656.5 LE, which is close to a previously reported value of $800,000 [36]. A decrease in milk production due to subclinical mastitis has been proven to have a great effect economically [37,38]. The SCM production losses are calculated based on the BTSCC. Reduced milk production is the largest indirect cost associated with mastitis, although the precise size of this loss varies [39-41]. The losses because of a high BTSCC can be considered invisible losses, so they are hidden costs or a lost opportunity for income [11]. The measurement of the SCC in bulk milk is the universal method of evaluating the occurrence of mastitis in dairy herds; therefore, there are significant correlations between the BTSCC of a farm and the economic losses related to decreased milk production and quality [11,42].

The results of quantifying the economic losses of CM for 80 CM cases from 20 dairy cattle farms revealed that the economic impact per case of CM could be classified into two main categories: Failure costs and preventive costs. Failure costs were, in turn, classified into two main categories: Direct and indirect costs. Direct costs include diagnostic testing, therapeutics, discarded milk, veterinary services, and labor. Indirect costs include future milk production loss and costs related to premature culling. Preventive costs are related to preventive measures in terms of equipment, consumables (diagnostics and chemicals), and the use of other resources to prevent diseases (increased labor) [43]. The total costs of 80 CM cases, including the failure and preventive costs, resulted in a total economic cost of 1,196,871.4 LE, including 1,169,150.4 LE for failure costs (106,336 LE for direct costs and 1,062,814.4 LE for indirect costs) and 27,721 LE for preventive costs, as shown in Table-1. This finding does not match the value of $19,889 [26], with an average cost per CM case of 28,760.9 LE. This result is discordant with previously reported values of $179 [10], $254 [11], $266 [12], $444 [13], €500 [44], $518 [14], €519 [5], and €269 [45].

The mean cost of veterinary time and consultation fees of CM was 250 LE per case. This result is discordant with previously reported values of $4 [13] and €54 [46]. The costs of veterinary time and consultation fees represent 1% of the total costs per clinical mastitis case, which agrees with a previous study [13], in which the cost of veterinary services was asserted to represent a small amount of the cost of each clinical mastitis case, but fails to match another reported value of 14% [46].

The mean cost of labor related to CM was 562.5 LE per CM case, which matches previously reported values of €24 [46] and $21 [13], but is discordant with another study [27], in which the labor cost per CM case was estimated at €82. Labor cost represents 2% of the total costs per case, which generally accords with the previous findings of 3% [26], 7% [46], and 4% [13]. In many countries, besides delivering drugs, the veterinarian might have to spend time on diagnosis of a CM case, so it is very difficult to estimate the cost of labor [47]. The cost of labor also varies from one farm to another. The problem with quantifying labor is in determining the hourly price; the workers’ time should also be included in the calculations of the cost of mastitis. The assessed time spent per CM case varies and depends on various factors such as the type of mastitis, milk yield, farm size, hired labor, and farm owner. For example, acute cases of mastitis, characterized by general illness, require more time for treating, nursing, and frequent stripping than mild sub-acute mastitis, which is associated only with changes in the milk [24].

Indirect CM costs such as premature culling costs are complicated to determine [7]. The cost of premature culling due to CM was 736,000 LE for 80 CM cows per year. The total number of cullings at 20 cattle dairy farms with 1178 cows was 34 cows (2.88%). Our findings agree with corresponding previous values of 15% [27] and 20% [11], while being discordant with others of 16% [48] and 9% [49]. Culling is the result of a decision by the dairy farmer. Future culling and replacement loss represents 23,000 LE or 77% of the total cost of a case of CM. Our results agree with the previous studies [7,24,50,51], in which it was mentioned that cows with mastitis have a higher risk of being culled and, therefore, the premature culling cost is considered one of the most important components of the economic costs of mastitis CM is one of the foremost important reasons for culling. Our results fail to match previous findings of €72 (20%) [46] and 39.4% [51], and a previous study [13], in which it was reported that future culling and replacement loss represents $182 or 41% of the total cost of a case of CM, or 48% [26] of this cost. It is vital to understand the culling process in dairy herds and its consequences so as to optimize dairy production [52]. Involuntary culling can be defined as when a cow leaves the herd for reasons that do not seem to be of the farmer’s choice. Udder health is considered one of the foremost reported reasons for culling in dairy herds [53]. Culling behavior by producers is additionally highly dependent on and determined by farms’ specific management factors (stocking density, disease incidence, disease detection, and treatment success) and also on the economic climate at the time (feed costs, availability and cost of replacements, and cow prices on the market) [54]. Cows that do not respond favorably to treatment can have repeated flare-ups of CM and should be culled, as their continued presence within the herd may result in other cows becoming infected. The cost related to involuntary culling because of CM is taken into account among the most important parameters and constituents of the total cost of CM and is known as a hidden cost that is not obvious to dairy farmers [6]. Further economic losses will be expected as the milk yield of primiparous cows is lower than that of multiparous cows, and since that yield of a heifer is also disappointing [7]. Culling cost is very complicated to determine. We calculated the difference between the price of a dairy cow for milk production and the price of a cow when culled for meat at a slaughterhouse. This reached about 23,000 LE, which matches a previous finding of €1051 [55], so the decision to cull a cow with mastitis can be seen as a loss, but may be seen as a preventive measure against persistent, recurrent mastitis to prevent new cases of mastitis within the herd, providing an additional benefit in agreement with a previous study [11]. Culling might play a very important role in reducing the overall herd-level prevalence of CM [6]. Culling of cows with chronic mastitis is commonly considered an effective method for controlling mastitis [56].

The total economic loss due to decreased milk production as a result of CM for 80 CM cases was 326,814 LE, which does not match a previous finding of $6,703 [26]. Decreased milk production as a result of CM represents 4085.18 LE of the total cost of a CM case. Our results match a previous value of €132 [46] but not values of €64 [57] and $115 [10]. The total economic losses due to decreased milk production as a result of CM represent 13.7% of the total cost of clinical mastitis cases; this result almost matches a previous finding of 18.2% [58] but is largely discordant with values of 25.5% [57], 28% [13], 34% [26], 64% [10], and 62.18% [59]. Out of the total of 80 CM cases from 20 Egyptian dairy farms, 27 animals at first parity showed a decrease in milk production (17%), while 53 animals at second parity or later showed a 22% decrease in milk production. These results fail to match those of a previous study [60], in which it was mentioned that the milk loss in the 1^st^ month after CM was assumed to be reduced by 40% in primiparous and 50% in multiparous cattle. The second parity multiparous cows are at a higher risk of developing CM (277,541.71 LE) and suffer more severe milk yield loss than primiparous cows (49,272.96 LE), which agrees with the previous findings [61]. The economic losses due to decreased milk production related to CM are regarded as one of the most important components of the cost of CM [11].

The total cost of CM (expenditures) extra control and preventive measures, including the diagnosis of CM per 80 cows annually in 20 Egyptian dairy farms, was 27,721 LE, which was lower than the milk yield economic costs. (Expenditures) extra control and preventive measures represented 346.5 LE per clinical case, which generally matches a previous finding of €15 [46]. (Expenditures) extra control and preventive measures represented 1% of the total cost of a case of CM. Mastitis preventive costs were composed mainly of costs related to farm management measures implemented to prevent mastitis. The preventive costs of every measure generally consisted of three main cost factors: Labor, consumables, and investments. Labor is defined as the time necessary to perform the measure. Consumables consist of measures of used goods. Investments were the depreciation and deflation costs of materials lasting longer than a year [27]. Our findings match a previous study [26], in which it was stated that the costs of mastitis preventive and control measures on Canadian dairy farms were lower than the milk yield costs, but not another study [58], in which the cost of mastitis preventive and control measures on Canadian dairy farms was estimated at €120 per cow per year, which was higher than the milk yield costs. We take into consideration the most important expenditures and control measures performed for the control of CM; these include cleaning of cubicles, pre- and post-milking teat dipping, use of gloves for milking, dry cow therapy, and maintenance of milking machines. There is also a substitution relationship between preventive costs and failure costs. The higher the preventive costs, the lower the failure costs of production diseases, and vice versa [43]. Studies on the economics of mastitis have generally dealt with failure costs. Only a few studies have assessed the economic losses of preventive measures. The costs of pre-milking teat dipping include the time the milker takes for pre-stripping, washing, and drying the udders; the use of water and teat disinfectant for washing; and the use of paper towels for drying the udders [24]. Expenditure on mastitis control is determined, by which control methods are implemented, that is, pre-milking preparation of the udders, teat disinfection, dry cow therapy, monitoring measures, and maintenance of milking machines [24]. The cost of monitoring and diagnostic measures for 80 CM cases was 8635.2 LE, representing 107.94 LE or 1% of the total cost of a case of CM. Our results agree with one study’s finding of a corresponding value of 2% ($10) [13] but disagree with another of $59 [26]. The cost of detecting and characterizing mastitis-causing organisms from infected cows varies and depends on the number of samples submitted and the laboratory used for culturing. The price of materials (wipes, iodine, alcohol, and sample tubes) should be added to the calculations of the mastitis control program. The labor cost is known as an element of the mastitis cost and should also be added to the calculations.

The mean drug and treatment cost of the 80 cases of CM were 328.85 LE per case. Therapeutic costs, which are often the most visible costs to producers, represented only 1% of the total costs of a case of CM. Our results disagree with a value of 23% in one study [62] and findings in another study [13], in which the cost of treatment of clinical cases was estimated to be $36, representing 8% of the total cost of a CM case. Treatment of cows suffering from SCM during the milking period is infrequently performed in Egypt because of concerns regarding the economic efficiency of SCM treatment during lactation and the risk of antimicrobial residues [63]. Therapeutic protocols are based mainly on the severity of clinical signs, and on many farms, not all CM cases are treated [26]. Different treatment protocols were thus known to be used for mild and moderate CM compared with severe CM [26]. When mild and moderate CM is treated, such cases are supposed to be treated only with intramammary antimicrobials. The treatment of severe CM, based mainly on systemic antimicrobials and anti-inflammatory drugs in addition to the typical intramammary treatment. The therapeutic costs of CM depend mainly on the prognosis of CM cases. Based on the assumption that discarded milk was fed to calves, the results in the tables revealed that the mean CM-related cost of discarded milk was 185.25 LE. The cost of discarded milk due to CM represented only 1% of the total costs of a case of CM. This finding nearly agrees with a value of $25 (5.7%) [13] but disagrees with ones of $1445 (11%) [26], 60% [10], 10% [11], €60 [46], and 73% [62]. The amount of discarded milk is the cow’s daily production at the time of onset of clinical symptoms or treatment and is usually multiplied by 6 days in terms of the milk price given to the farmer. In some farms, some discarded milk could be used as calf feed. The choice of feeding mastitic milk to calves might offer an annoyed farmer some support, but should be carefully considered.

## Conclusion

This study quantified the cost of clinical and subclinical mastitis in cattle using average linear scores [found on The DHIA SCC sheets] and BTSCC per lactation number. It gives insight into current market conditions and management practices in Egypt. The prevalence of cattle mastitis (CM and SCM) in individual cows at the examined dairy farms was 57.1% (240/420). CM was found in 19% of cows (80/420). The analysis of BTM samples for SCC revealed that the geometric mean of SCCs/ml in BTM samples at 150 cattle dairy farms was 565.3×10^3^. The results of analyzing the economic losses due to SCM revealed that the annual SCM economic loss was 21,933,258.6 LE and that the two most important cost components were the subsequent decrease in milk production and quality premium losses (93% and 7% of SCM costs, respectively). The total quality premium loss for 150 cattle dairy herds with a total of 8867 cows was 1,369,602.12 LE. On the other hand, the annual economic loss due to decreased milk production as a result of SCM was 20,563,656.5 LE. In terms of the total cost of 80 CM cases including the failure and preventive costs, a value of 1,196,871.4 LE was obtained, including 1,169,150.4 LE failure costs (106,336 LE for direct costs and 1,062,814.4 LE for indirect costs) and 27,721 LE preventive costs, with an average cost per CM case of 28,760.88 LE. The mean cost of veterinary time and consultation fees represented 250 LE (1%) per CM case. The mean cost of labor was 562.5 LE (2%) per CM case. The cost of premature culling due to CM was 736,000 LE per 80 CM cows per year, and the total percentage of culled animals in 20 cattle dairy farms with 1178 cows was 20.8%. Future culling and replacement loss represented 23,000 LE or 77% of the total cost of a case of CM. The total economic loss due to decreased milk production as a result of CM for 80 CM cases was 326,814 LE. Decreased milk production as a result of CM was represented 4085.18 LE or 13.7% of the total cost of a clinical mastitis case. The total cost of CM (Expenditures) extra control and preventive measures, including the diagnosis of CM in 80 cows annually in 20 Egyptian dairy farms, was 27,721 LE, representing 346.5 LE or 1% of the total cost of a CM case. The cost of monitoring and diagnostic measures for 80 CM cases was 8,635.2 LE, representing 107.94 LE or 1% of the total cost of a case of CM. The mean drug and treatment costs of CM were 328.85 LE per CM case. Therapeutic costs represented only 1% of the total costs of a case of CM. Based on the assumption that discarded milk was fed to calves; the mean discarded milk cost of CM was 185.25 LE. The cost of discarded milk of CM represented only 1% of the total cost of a case of CM. The method used for cost estimation in this study is highly adaptable to individual cattle farms and played a major role in assessing specific control and management measures. The concepts described in this study help to improve our understanding of the full economic impact of clinical and subclinical mastitis in cattle.

## Authors’ Contributions

MFA and HMY designed the concept of the review article, and MFA and HMY designed and performed study design and the economic framework. SAE drafted and revised the manuscript. All authors read and approved the final version.
